# PB.13. Impact on film reading of moving from analogue to digital screening: a service evaluation - South East Scotland Breast Screening Programme

**DOI:** 10.1186/bcr3705

**Published:** 2014-11-03

**Authors:** G Babu, A Gilchrist, J Murray

**Affiliations:** 1 NHS Lothian, Edinburgh, UK

## Introduction

In 2013 this unit became the first in Scotland to switch from analogue to digital mammographic screening. This study analyses the impact of this change on film reading practice with a reflective questionnaire and comparative analysis of recall, arbitration rates and positive predictive values (PPV).

## Methods

Film readers in this unit are radiologists, radiographers and clinicians. A questionnaire was designed to assess individual reading practice before and after the switch to digital screening including perception of lesion types. We used SBSP reports and internal statistics for the comparative analysis.

## Results and conclusion

Eleven of 13 film readers responded. All readers agreed they enjoyed digital reading. All readers agreed the ability to manipulate images was useful. Most readers felt they would have liked more training when digital mammography was introduced. Eight readers felt digital was convenient and easier to read. Three felt it took longer. Several recorded intermittent eye strain. Readers found comparison with previous analogue films problematic. All readers review their reading figures annually. Nonradiologists do formalised self-audit, which continues after training. This tool is less used by radiologists, who obtain informal feedback from clinics and MDM. Fifty per cent of readers felt that reading guidelines would be helpful. With digital reading, all were confident reading opacities but recalling more calcifications. Readers were generally less confident with asymmetries and parenchymal distortions. Using two comparable 6-month periods, recall rates showed an initial peaking with introduction of digital screening. Arbitration rates increased but PPV significantly improved.

